# The association between ambient fine particulate air pollution and physical activity: a cohort study of university students living in Beijing

**DOI:** 10.1186/s12966-017-0592-x

**Published:** 2017-10-05

**Authors:** Hongjun Yu, Miao Yu, Shelby Paige Gordon, Ruiling Zhang

**Affiliations:** 1 Department of Physical Education, Tsinghua University, Tsinghua Yuan Str, Beijing, 100084 China; 2 Department of Reference Service in Library, Renmin University of China, Zhongguan Cun Str, Beijing, 100872 China; 3 Department of Interdisciplinary Health Sciences, University of Illinois at Urbana-Champaign, Urbana, IL USA

**Keywords:** Air pollution, Fine particulate matter, Physical activity, Moderate-to-vigorous physical activity, youth

## Abstract

**Background:**

Air pollution has become a substantial environmental issue affecting human health and health-related behavior in China. Physical activity is widely accepted as a method to promote health and well-being and is potentially influenced by air pollution. Previous population-based studies have focused on the impact of air pollution on physical activity in the U.S. using a cross-sectional survey method; however, few have examined the impact on middle income countries such as China using follow-up data. The purpose of this study is to examine the impact of ambient fine particulate matter (PM_2.5_) air pollution on physical activity among freshmen students living in Beijing by use of follow-up data.

**Methods:**

We conducted 4 follow-up health surveys on 3445 freshmen students from Tsinghua University from 2012 to 2013 and 2480 freshmen completed all 4 surveys. Linear individual fixed-effect regressions were performed based on repeated-measure physical activity-related health behaviors and ambient PM_2.5_ concentrations among the follow-up participants.

**Results:**

An increase in ambient PM_2.5_ concentration by one standard deviation (44.72 μg/m^3^) was associated with a reduction in 22.32 weekly minutes of vigorous physical activity (95% confidence interval [CI] = 24.88–19.77), a reduction in 10.63 weekly minutes of moderate physical activity (95% CI = 14.61–6.64), a reduction in 32.45 weekly minutes of moderate to vigorous physical activity (MVPA) (95% CI = 37.63–27.28), and a reduction in 226.14 weekly physical activity MET-minute scores (95% CI = 256.06–196.21). The impact of ambient PM_2.5_ concentration on weekly total minutes of moderate physical activity tended to be greater among males than among females.

**Conclusions:**

Ambient PM_2.5_ air pollution significantly discouraged physical activity among Chinese freshmen students living in Beijing. Future studies are warranted to replicate study findings in other Chinese cities and universities, and policy interventions are urgently needed to reduce air pollution levels in China.

## Background

Air pollution is a major environmental issue affecting human health worldwide. According to the WHO, ambient air pollution may have contributed to approximately 4.2 million deaths worldwide in 2015 [1]. One type of ambient air pollution that has received increasing attention is fine particulate matter, specifically PM_2.5._ Particulate matter (PM) is mainly derived from industrial activities such as power generation, which is the combustion of coal and fossil fuels produced when heated [2]. PM is a mixture of solid and/or liquid particles suspended in the air. PM_2.5_ refers to particles that are less than 2.5 μm micrometers in diameter. PM_2.5_ can be effectively inhaled and deposited in the airway and alveolar surfaces, causing health problems [3]. Previous studies have shown that adverse health effects of short- and long-term exposure to PM_2.5_ include blood pressure, myocardial infarction, stroke and all-cause mortality [4–7].

Physical inactivity is a leading risk factor of non-communicable disease (NCD). The WHO reports that it is the fourth leading cause of global mortality and leads to 3.2 million deaths each year [8]. Regular physical activity has many health benefits, including reduced all-cause mortality and reduced chronic disease such as cardiovascular diseases, diabetes, colon and breast cancer, hypertension, obesity and depression [9]. The WHO recommends that adults participate in at least 150 min per week of physical activity at a moderate-to-vigorous (MVPA) intensity [10]. MVPA has significant health benefits for youth and adults, including preventing obesity and decreasing blood pressure [11–13]. The U.S. Department of Health and Human Services recommends that youth accumulate ≥60 min per day of MVPA to prevent obesity and to benefit their physical fitness, bone health, metabolic and cardiovascular health [14]. However, it is estimated that 23% of adults and a vast majority of adolescents and youth fall short on the recommended guidelines for MVPA per week [15–18].

Although the adverse effects of air pollution and physical inactivity on health risks have been well studied, little knowledge is known regarding air pollution impact on physical activity. Air pollution may dismiss people from engaging in regular physical activity through several mechanisms. Air pollution has been associated with respiratory diseases, such as asthma and bronchitis [2, 19], resulting in impaired exercise performance [20]. The presence of smog can also restrict people from engaging in outdoor physical activity [21]. Media alerts of air quality to inform the public about harmful air pollution may affect people’s physical activity behavior [22]. A few studies have linked air pollution to a decrease in exercise performance in athletes [23, 24], but little previous research has reported the relationship between air pollution and physical activity based on population-based evidence. To our knowledge, a total of 4 population-based studies have estimated the relationship between air pollution and physical activity [21, 25–27]. Previous studies have mainly analyzed survey data from the Behavioral Risk Factor Surveillance System (BRFSS), a large cross-sectional survey of U.S. adults 18 years of age and older [21, 25, 26]. Air pollution contribution (PM_2.5_) was consistently found to be associated with a modest, but measurable, decrease in individuals’ leisure-time physical activity [21, 25, 26].

Despite the aforementioned work, two major gaps in the scientific literature remain. Previous studies have exclusively focused on the impact of air pollution on physical activity in the U.S., but few studies have examined middle-income countries such as China. In the U.S., most counties have already met the national air quality standards levels. There are less than 10 counties in the U.S. that do not meet the new national air quality standard in reaching 12.0 μg/m^3^ by 2020 [28]. Since starting in the 1980’s, China has experienced rapid industrial and urban development. As a result, air pollution has become a substantial threat to public health in China, the largest middle-income country in the world. In 2010, air pollution in China was the fourth cause of mortality and led to 1.2 million premature deaths, almost 40% of the global total [29]. Beijing, the capital city of China, has been experienced severe air pollution, with the highest annual average level of PM_2.5_ exceeding 600 μg/m^3^ [3]. Yet, no similar relationship between air pollution and physical activity has been explored among young adults. Previous studies have analyzed cross-sectional data that was subject to confounding bias due to unobserved differences in individual characteristics. Behavioral change in response to temporal variations in air quality could not be assessed due to data unavailability. The only other middle-income country-based study available examined the influence of PM_2.5_ air pollution on health behaviors among university retirees in China [30]. High PM_2.5_ concentration was found to discourage retired older adults from engaging in daily physical activities [30]. The strength of this study was in its prospective design; however, this was limited by convenience sampling which comprised its external validity [27]. There are currently no follow-up large sample studies on examined the influence of PM_2.5_ air pollution on physical activity among youth populations.

The purpose of this study is to examine the follow-up relationship between ambient PM_2.5_ air pollution and behavioral outcomes pertaining to weekly physical activity levels among university freshmen in Beijing. We hypothesized that in response to elevated air pollution, study participants reduced their physical activity.

## Methods

### Participants

A paper-based health survey was conducted on a regular basis during students’ freshman year at Tsinghua University. The survey was administered in class by faculty and all freshmen participants took the survey within a one-week health education class window (every other day, Mon to Fri). Survey participation was voluntary. Upon signing the consent form, survey participants were asked to complete a paper-based questionnaire and hand it in after the course. The survey included questions regarding one’s sociodemographic, physical and mental health status, and health and risk behavior. The same survey was administered four times within the freshman year of newly admitted undergraduate students entering the university in 2012 (September 24–28, 2012; November 5–9, 2012; March 5–9, 2013; May 13–17, 2013). Survey participants were asked to report their student identification number and that information was used to link multiple survey questionnaires completed by the same respondent.

**Fig. 1:**
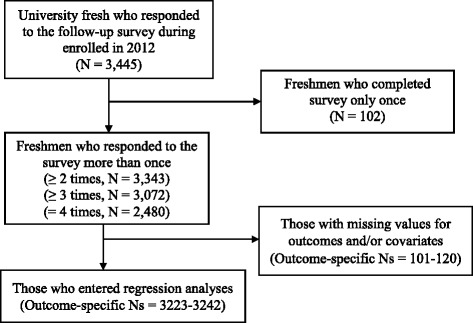
Study Sample Flowchart

Figure 1 presents the analytic sample selection flowchart for the 4 follow-up surveys administered. A total of 3445 Tsinghua University freshmen enrolled in 2012 responded to the survey. Among them, 3343 participated in more than one survey, 3072 participated in 3 or more surveys and 2480 participated in all 4 surveys. Among those repeated respondents, 3223 to 3242 (dependent upon specific outcome variables) had non-missing values for the specific outcome and all covariates, therefore, were included in the regression analyses. The study was approved by the Tsinghua University Institutional Review Board (IRB#2012534001).

### Environmental measures

Environmental measures included average ambient PM_2.5_ concentration (μg/m^3^), average daytime temperature (C), average wind speed (m/s) and percentage of rainy days in Beijing, China over the last seven days before the health survey was administered. Hourly ambient PM_2.5_ concentration data came from the Mission China Air Quality Monitoring Program run by the U.S. Department of State (September 17th 00:00 - 27th 23:00, 2012; October 29th 00:00 – November 8th 23:00, 2012; February 25th 00:00 – March 8th 23:00, 2013; May 6th 00:00 – 16th 23:00, 2013). Daily weather data including daytime temperature, wind speed and precipitation came from the China Meteorological Administration.

To facilitate result interpretation, we standardized average ambient PM_2.5_ concentration over the last seven days through centering (i.e., subtracting the mean from each value) and then dividing by its own standard deviation (i.e., PM_2.5_ z-scores). The estimated coefficient of ambient PM_2.5_ concentration can thus be interpreted as the change of an outcome variable (e.g., total minutes of walking in the last week) with respect to a change of ambient PM_2.5_ concentration by one standard deviation (44.72 μg/m^3^).

### Physical activity measurements

The short version of the International Physical Activity Questionnaire (IPAQ) was used to measure physical activity (PA). The short version (9 items) of the IPAQ has been validated in China [31–33]. Data from the short IPAQ provided information on the time spent walking and on moderate- and vigorous-intensity activities.

Total minutes of vigorous physical activity in the last week were constructed based on the answers to two questions adapted from the International Physical Activity Questionnaire (IPAQ) [34]: “During the last seven days, on how many days did you do vigorous physical activities such as aerobics, running, fast bicycling and fast swimming?” and “How much time did you usually spend doing vigorous physical activities on one of those days?”. Total minutes of vigorous physical activity in the last week were calculated through multiplying daily average number of minutes spent on vigorous physical activity by its corresponding number of days.

Total minutes of moderate physical activity in the last week were constructed based on the answers to two questions adapted from the International Physical Activity Questionnaire (IPAQ) [34]: “During the last seven days, on how many days did you do moderate physical activities, such as carrying light loads, bicycling at a regular pace and playing doubles tennis?” and “How much time did you usually spend doing moderate physical activities on one of those days?”. Total minutes of moderate physical activity in the last week were calculated through multiplying daily average number of minutes spent on vigorous physical activity by its corresponding number of days.

Total minutes of walking in the last week were constructed based on the answers to the two questions adapted from the IPAQ [34]: “During the last seven days, on how many days did you walk for at least 10 minutes at a time?” and “How many minutes did you usually spend on one of those days walking?”. Total minutes of walking in the last week were calculated through multiplying daily average minutes spent walking by its corresponding number of days.

Total weekly minutes of MVPA were calculated by weekly average of minutes spent on moderate physical activity plus weekly average of minutes spent on vigorous physical activity. Data collected using the IPAQ were used to estimate weekly total physical activity in metabolic equivalent (MET) energy expenditure. A MET-minute score is computed by multiplying the MET score by the minutes performed [35].

### Statistical methods

Descriptive statistics including means, standard deviations and percentages were used to summarize and compare characteristics of the overall sample by sex. Chi-square tests were conducted to compare categorical variables and t-tests for continuous variables. These were repeat measures one-way ANOVA with Bonferoni(B) tests were conducted to compare the differences between the 4 follow-up surveys. Linear individual fixed-effect regressions were performed based on the repeated-measure survey data from freshmen student cohorts enrolled at Tsinghua University in 2012. The five continuous outcome variables were total minutes of vigorous physical activity, total minutes of moderate physical activity, total minutes of MVPA, total minutes of walking and total physical activity (MET-minute scores) in the week before the survey was administrated. The key independent variable in all regressions was the standardized average ambient PM_2.5_ air pollution concentration during the seven days prior to the survey. All models were controlled for the aforementioned individual-level time-variant covariates as well as the environmental measures: average daytime temperature, average wind speed and percentage of rainy days in the last seven days. Separate regressions were conducted for each outcome variable based on samples stratified by sex (i.e., entire sample with both gender, male only and female only).

Compared to the conventional pooled cross-sectional regression, individual fixed-effect regression is preferred. Individual fixed-effect regression only uses within-individual variations in physical activity level to identify the impacts of air pollution concentration, thus removing potential omitted variable bias due to differences in time-invariant individual characteristics such as genes, sex, ethnicity, habits and personal preferences. Due to the exclusive dependence upon within-individual variations in an outcome measure, individual fixed-effect regressions could only estimate the effect of a time-variant independent variable. Thus, time-invariant individual characteristics such as sex and ethnicity were not examined.

All statistical procedures were performed in Stata 14.2 SE version (StataCorp, College Station, TX). Eicker-Huber-White sandwich estimator was used to estimate the standard errors of regression coefficients, which addressed within-individual serial correlations.

### Individual-level covariates

The following individual-level time-variant covariates were controlled for in the regression analyses: a continuous variable for age in years, a continuous variable for body mass index (BMI; kg/m^2^) calculated from self-reported height and weight, a dichotomous variable for current smoking status (current non-smokers as the reference group), a dichotomous variable for current drinking status (current non-drinkers as the reference group), a continuous variable for self-rated physical health (on a scale of 1 to 10, worst to best) and a continuous variable for self-rated mental health (on a scale of 1 to 10, poor to excellent).

## Results

### The characteristics of survey participants

Table 1 summarizes baseline characteristics of the survey participants. A majority of the sample was composed of male participants (67.73%). Participants were mostly 18 years old (SD = 0.86), as they had just graduated from high school and entered Tsinghua University. The mean BMI was 21.21 kg/m^2^ (SD = 3.80). A rather small proportion of these freshmen were current smokers (0.78%) and drinkers (2.86%). Self-rated physical and mental health scores averaged 5.78 (SD = 1.82) and 7.06 (SD = 1.89), respectively. Survey participants on average engaged in 90.01 (SD =112.52) minutes of vigorous physical activity, 212.12 (SD = 173.76) minutes of moderate physical activity, 306.53 (SD = 228.99) minutes of MVPA, 71.61 (SD = 115.84) minutes of walking, 1723.45 (SD = 1372.42) total PA MET-minute scores, 8.94 (SD = 2.78) hours per day of sedentary behavior and 7.68 (SD = 0.83) hours per day of sleeping in the last week, respectively.

**Table 1:** Baseline characteristics of survey participants

	Total	Male	Female	*p*
N	3097	2070	1027	
*Age (yr), mean (SD)*	18.18 (.85)	18.22 (.89)	18.10 (.76)	0.000
*Ethnicity, n (%)*				
Han	2891 (90.43)	1953 (90.79)	938 (89.67)	0.313
Minority	306 (9.57)	198 (9.21)	108 (10.33)	
*Body mass index, mean (SD)*				
BMI (kg/m^2^)	21.21 (3.80)	21.82 (4.07)	19.98 (2.80)	0.000
*Smoking, n (%)*				
Current smoker	24 (0.78)	21 (1.01)	3 (0.30)	0.038
Current nonsmoker	3056 (99.22)	2065 (98.99)	991 (99.70)	
*Drinking n (%)*				
Current drinker	88 (2.86)	73 (3.50)	15 (1.51)	0.002
Current nondrinker	2992 (97.14)	2013 (96.50)	979 (98.49)	
*Self-rated physical health, mean (SD)*				
Physical health score (1–10)	5.78 (1.83)	5.77 (1.86)	5.80 (1.75)	0.687
*Self-rated mental health, mean (SD)*				
Mental health score (1–10)	7.07 (1.89)	7.09 (1.92)	7.02 (1.81)	0.339
*Leisure-time vigorous physical activity, mean (SD)*				
Total minutes of vigorous physical activity in last week	96.01 (112.52)	107.35 (115.65)	72.83 (102.04)	0.000
*Leisure-time moderate physical activity, mean (SD)*				
Total minutes of moderate physical activity in last week	212.12 (173.76)	219.63 (174.92)	197.52 (170.63)	0.001
*Leisure-time MVPA, mean (SD)*				
Total minutes of vigorous and moderate physical activity in last week	306.53 (228.99)	325.77 (231.13)	268.33 (219.89)	0.000
*Walking, mean (SD)*				
Total minutes of walking in last week	71.61 (115.84)	71.49 (110.14)	71.85 (126.42)	0.939
*Sedentary behavior, mean (SD)*				
Total hours of sedentary behavior per day	8.96 (2.78)	8.87 (2.80)	9.12 (2.75)	0.019
*Sleeping behavior, mean (SD)*				
Total hours of sleeping behavior per day	7.68 (0.85)	7.69 (0.84)	7.65 (0.87)	0.228
*Total MTEs in last week, mean (SD)*	1732.45 (1372.42)	1843.00 (1371.02)	1510.93 (1348.88)	0.000

### The relationship between air pollution and PA

**Fig. 2:**
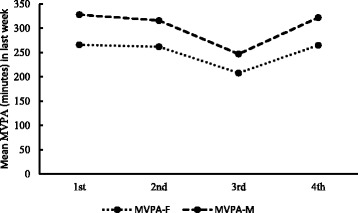
Mean of ambient PM_2.5_ air pollution (μg/m^3^) in last week of the survey during the period of the four times follow-up

**Fig. 3:**
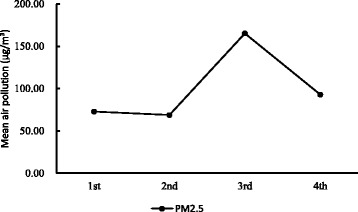
Mean of vigorous physical activity and moderate physical activity (MVPA) total minutes in last week during the four times follow-up survey. MVPA-F indicates the MVPA for female freshmen, MVPA-M indicates the MVPA for male freshmen

Table 2 shows the mean variations of environmental measures and physical activity of the freshmen in the four follow-up surveys. As shown in Table 2, PM_2.5_ mean measures results had significantly increased from 72.90 to 165.13 μg/m^3^ in Beijing over the last seven days before the surveys being administered (*p* < .001), and total minutes of MVPA in one week decreasing from 289.75 to 229.83 (p < .001). Figs 2-3 shows the variations of air pollution and MVPA in the four follow-up surveys by male and female. It demonstrates there were significant declining outcomes in MVPA with the ambient PM_2.5_ air pollution, increasing among the freshmen over the follow-up period. There were also large variations in the percentage of rainy days and large variations in daytime temperature and average wind speed across the survey periods.

**Table 2:** Average physical activity, air pollution concentrations and other environmental variables in the last seven days before survey

Survey order	1nd		2rd		3rd		4th	
*Dependent variable*								
Vigorous PA (weekly minutes), mean (SD)	95.81	(112.16)^**^	104.76	(90.62)^**^	77.18	(97.50)^***^	126.90	(104.29)^***^
Moderate PA (weekly minutes), mean (SD)	213.46	(175.24)^**^	195.64	(147.34)^***^	157.94	(131.37)^***^	177.35	(128.34)^***^
MVPA (weekly minutes), mean (SD)	289.75	(226.77)	286.96	(194.32)	229.83	(183.69)^***^	300.55	(188.60)
Walk PA (weekly minutes), mean (SD)	71.91	(115.73)	69.64	(112.51)	73.30	(102.43)^*^	81.05	(108.85)^**^
Sedentary behavior (hour/day), mean (SD)	8.96	(2.78)^***^	9.43	(2.91)^**^	9.21	(2.85)^*^	9.21	(2.92)^*^
Sleeping behavior (hour/day), mean (SD)	7.68	(0.85)	7.66	(0.91)	7.87	(0.86)^***^	7.79	(0.99)^***^
Total Met, mean (SD)	1835.67	(1381.20)	1848.72	(1221.28)	1501.97	(1151.37)^***^	1994.27	(1228.22)^***^
*Independent variables*								
PM2.5 (μg/m^3^), mean (SD)	72.90	(50.26)	68.78	(58.48)	165.13	(131.98)^***^	92.88	(70.43)^*^
*Covariates variables*								
Temperature (°C), mean (SD)	25.36	(2.87)	11.64	(4.20)^***^	12.17	(5.13)^***^	28.73	(3.00)
Wind (m/s), mean (SD)	3.23	(0.75)	3.55	(0.65)	3.38	(1.00)	3.41	(0.44)
Rain %, mean (SD)	0.36	(0.51)	0.18	(0.41)	0.08	(0.29)	0.18	(0.41)

Results of repeated measures one-way ANOVA analysis. ^*^ *P* < 0.05; ^**^ *P* < 0.01; ^***^ *P* < 0.001

### Impact of air pollution on PA

Table 3 reports the estimated effects of PM_2.5_ concentrations on individual-level outcomes pertaining to physical activity level using linear individual fixed-effect regressions. PM_2.5_ concentrations were found to be negatively associated with total minutes of physical activity in the last week among survey participants. Specifically, an increase in PM_2.5_ concentrations by one standard deviation (i.e., 44.72 μg/m^3^) was associated with a reduction in weekly total minutes of vigorous physical activity by 22.32 (95% confidence interval [CI] = −24.88, −19.77), a reduction in weekly total minutes of moderate physical activity by 10.63 (95% CI = −14.61, −6.64), a reduction in weekly total minutes of MVPA by 32.45 (95% CI = −37.63, −27.28) and a reduction in weekly total physical activity MET-minute scores by 226.14 (95% CI = −256.06, −196.21). Ambient PM_2.5_ concentration was not associated with minutes spent walking by the freshmen (*p* > 0.05).

**Table 3:** Estimated effects of PM_2.5_ on individual-level physical activity outcomes by sex

Dependent variable	Total		Male only		Female only	
	Coefficient (95% CI)	# Observations (# participants)	Coefficient (95% CI)	# Observations (# participants)	Coefficient (95% CI)	# Observations (# participants)
Total minutes of vigorous physical activity in last week	−22.32^***^ (−24.88, −19.77)	11,318 (3241)	−21.70^***^ (−24.94, −18.46)	7654 (2189)	−23.55^***^ (−27.59, −19.52)	3664 (1065)
Total minutes of moderate physical activity in last week	−10.63^***^ (−14.61, −6.64)	11,045 (3231)	−13.27^***^ (−18.24, −8.29)	7416 (2181)	−4.79 (−11.41, 1.83)	3629 (1063)
Total minutes of MVPA	−32.45^***^ (−37.63, −27.28)	10,832 (3228)	−34.72^***^ (−41.22, −28.23)	7297 (2179)	−27.29^***^ (−35.75, −18.83)	3535 (1062)
Total minutes of walk in last week	−.86 (−3.84, 2.12)	11,018 (3233)	−2.68 (−6.32, .95)	7411 (2183)	3.30 (−1.91, 8.52)	3607 (1063)
Total met in last week	−226.14^***^ (−256.06, −196.21)	11,589 (3242)	−238.10^***^ (−275.72, −200.47)	7809 (2190)	−198.19^***^ (−246.91, −149.46)	3780 (1065)

Separate individual fixed-effect regressions were performed to estimate the effects of air pollution concentrations on samples stratified by sex

Models adjust for all time-variant individual characteristics listed in Table 1 (i.e., age, BMI, smoking status, drinking status, self-rated physical health, and self-rated mental health) and environmental variables listed in Table 2 (average daily temperature, average wind speed, and percentage of rainy day in last week). ^*^ *P < 0.05;* ^**^ *P < 0.01;* ^***^ *P < 0.001*

The estimated effects were similar between the male and female students except for weekly total minutes of moderate physical activity. An increase in ambient PM_2.5_ concentration by one standard deviation was associated with a statistically significant reduction in weekly total minutes of moderate physical activity by 13.27 (95% CI = −18.24, −8.29) among males (*p* < .001); whereas the estimated decline by 4.79 (95% CI = −11.41, −1.83) in weekly total minutes of moderate physical activity in response to an elevated PM_2.5_ concentration level was much smaller and statistically nonsignificant for females (p > 0.05).

Regarding other environmental measures and individual-level covariates (results not shown in table), wind speed and percentage of rainy days over the last seven days were consistently negatively associated with physical activity, but positively associated with daily average daytime temperature. High self-rated physical health was positively associated with physical activity and high self-rated mental health was positively associated with physical activity over in the past week.

## Discussion

This study assessed the longitudinal relationship linking ambient PM_2.5_ concentration to behavioral modifications pertaining to weekly physical activity level among university freshmen in Beijing from 2012 to 2013. Ambient PM_2.5_ concentration was found to be negatively associated with weekly total minutes of physical activity behavior among survey participants. The impact of ambient PM_2.5_ concentration on weekly total minutes of moderate physical activity tended to be greater among males than females. To our knowledge, this study is the first known cohort study to examine the impact of air pollution concentration on young adults’ health behaviors in a middle-income country. This study reveals that air pollution leads to a decrease in MVPA behavior in young adults in China.

In our study, ambient PM_2.5_ was negatively associated with physical activity in Chinese youth. Our results suggest an increase in ambient PM_2.5_ dismisses physical activity among freshmen. This finding is consisted with previous research [21, 25, 26] assessing the relationship between air pollution and physical inactivity, which finds that an increase PM_2.5_ leads to the increasing prevalence of physical inactivity. These three studies all found that air pollution may negatively influence physical activity behavior; moreover, the air pollution problem in China has attracted worldwide attention in recent years. Previous studies have reported that air pollution, especially ambient air pollution, dismiss outdoor physical activity among children and adolescents in China [36, 37], which is a distinctive public health challenge in China [38]. In our study, we found that there was a significantly declining impact of ambient PM_2.5_ on vigorous physical activity, which is inconsistent with previous results [39]. A recent study in Beijing of 40 Han Chinese participants found that there was no impact of ambient PM_2.5_ and ambient temperature on physical activity [39]. A possible explanation for this difference could be that 40 participants is too small of a sample and may have been subjected to social behavior desirability bias [40].

The WHO guideline recommends that adults need a minimum of 150 min of moderate-to-vigorous physical activity (MVPA) per day [41]. Our finding shows that an increase in PM_2.5_ concentration significantly discourages Chinese freshmen students from performing more than 30 min of MVPA behavior per week, less than 20% of the current guideline. MVPA is considered as an effective intervention to promote health in youth [42]. Previous studies show that MVPA can help prevent weight gain and maintain a healthy body weight in youth [43]; however, physical inactivity and sedentary behavior are growing problems among Chinese youth [44]. The prevalence of obesity among Chinese youth more than doubled, from 1993 (6.1%) to 2009 (13.1%) [45]. According to the China Health and Nutrition Survey, the most common forms of MVPA among Chinese youth are performed outdoors [46]. Exposure to air pollution and outdoor physical activity among Chinese youth has become a serious public health problem [36]. Our result provides the new evidence that air pollution dismisses Chinese youth from performing MVPA behavior, thus exposing them to elevated health risks.

Previous studies [47, 48] showed that other weather-related variable conditions, such as temperature, strong wind, and rain, may also play important roles in influencing physical activity levels. This finding was consistent with several previous publications that found that wind speed and percentage of rainy days were consistently negatively associated with physical activity, but positively associated with daily average daytime temperature. For example, individuals may decrease MVPA when temperature is too cold. In the winter in Beijing, it was much colder (average 12 °C) than in the summer (average 25 °C). At the same time, the air pollution was much worse in the winter than in the summer [49].

Our finding indicates significant reductions in physical activity relative to ambient PM_2.5_ level. In our study analysis, a change in ambient PM_2.5_ concentration by one standard deviation (44.72 μg/m^3^) is similar to the government air quality one cut off standard (50 μg/m^3^) threshold. There are six thresholds for air pollution in China [2]: 0-50 μg/m^3^ (good, green warning), 51-100 μg/m^3^ (fair, yellow warning), 101-150 μg/m^3^ (mild pollution, orange warning), 151–200 (moderate pollution, red warning), 201-300 μg/m^3^ (severe pollution, purple warning) and >300 μg/m^3^ (serious pollution, maroon warning). It is interesting to note that in our study, ambient PM_2.5_ was in the yellow warning level during the first, second and forth follow-up survey, the freshmen’s physical activity level did not decrease in a linearly regression with an increase in PM_2.5_ value. When ambient PM_2.5_ was in the red warning level during the third follow-up period, participants’ physical activity decreased rapidly. A potential explanation for the difference in air pollution level contributors in to decreasing physical activity is that smog air, warning guidelines, and media alerts create a combined effect in dismissing people from performing physical activity, especially in outdoor exercise and vigorous physical activity. Consistent with previous research, media alert perceptions of air pollution were statistically significantly associated with physical activity behavior in adults [22].

Subgroup analyses suggested that male’s moderate physical activity patterns tended to be more responsive to the variations in ambient PM_2.5_ concentration than female’s. This could be partially explained by the Chinese gender perception difference of bad air quality risk. Compared to males, females tend to perceive poor air quality as a health risk and may reduce their risk of exposure by changing or reducing their outdoor activity. However, our findings on the sex differences among youth are rather preliminary and warrant replication by future studies.

The strengths of this study lie in its longitudinal study design, reliable and time-sensitive environmental measures and a comprehensive sample of 4 waves of follow-up for all freshmen at Tsinghua University from 2012 to 2013. Nevertheless, a few major limitations of this study should be noted. The sample was from only one university. Freshmen cohorts from one university are unlikely to represent the entire undergraduate population in Beijing, or nationwide, which confines the generalizability of the study findings. Future follow-up studies with representative samples are warranted to replicate findings of this study and produce generalizable estimates. Health behaviors were all self-reported and subject to recall error and social desirability bias [50]. The Mission China Air Quality Monitoring Program led by the Ministry of Environmental Protection of the People’s Republic of China had data air pollution dating back to December 2013, but did not provide any data earlier than this time. The U.S. Department of State published data on air pollution in 2011 but only had ambient PM_2.5_ data. Beijing covers a rather large metropolitan area and different neighborhoods may have different levels of air pollution concentration. Using the city-average air pollution measure could have masked considerable local variations. The individual fixed-effect models eliminated confounding bias from factors that remained constant within-participant over time, but it did not control for more transient unobserved factors such as daily variations in pains and emotions. Moreover, it is the increase in potential error by conducting multiple regression analyses with highly correlated dependent variables. Previous studies have shown that there is a declining trend of physical activity behavior among freshmen university students as they progress through their first academic year, even without exposure to air pollution [51]. Therefore, this is a significant confounder, and future follow-up studies should control the confounder and figure out a clearer relationship between air pollution and physical activity behavior among freshmen university students. Additional limitations of the present study include the following: it is not known if individuals are exercising indoors or outdoors, changes in physical activity participation may not vary by air pollution if an individual is active indoors, heavy air pollution has gradually become a norm that residents in many large Chinese cities have to live with, and switching from outdoor physical activities (eg, running on sidewalks) to indoor exercises (eg, running on a treadmill) could partially offset the disruption of air pollution.

## Conclusion

This study examined the longitudinal relationship between air pollution concentration and behavioral modifications pertaining to weekly physical activity level among university freshmen in Beijing, China. Ambient air pollution concentration was negatively associated with weekly total minutes of physical activity behavior. Ambient PM_2.5_ plays a key role in discouraging youth from performing physical activity behavior. Future studies are warranted to replicate findings of this study in other Chinese cities and universities, and policy interventions are urgently called to reduce air pollution level in China.

## Abbreviations

BMI: Body mass index
MET: Metabolic equivalent
MVPA: Moderate-to-vigorous physical activity
PM2.5: Particulate matter with a diameter less than 2.5 μm
